# Marine Biocatalysts: Enzymatic Features and Applications

**DOI:** 10.3390/md9040478

**Published:** 2011-03-25

**Authors:** Antonio Trincone

**Affiliations:** Institute of Biomolecular Chemistry, National Research Council, Via Campi Flegrei, 34, 80078 Pozzuoli, Naples, Italy; E-Mail: antonio.trincone@icb.cnr.it; Tel.: +39-081-867-5095; Fax: +39-081-867-5095

**Keywords:** biocatalysis, marine enzymes, marine biocatalysts

## Abstract

In several recent reports related to biocatalysis the enormous pool of biodiversity found in marine ecosystems is considered a profitable natural reservoir for acquiring an inventory of useful biocatalysts. These enzymes are characterized by well-known habitat-related features such as salt tolerance, hyperthermostability, barophilicity and cold adaptivity. In addition, their novel chemical and stereochemical characteristics increase the interest of biocatalysis practitioners both in academia and research industry. In this review, starting from the analysis of these featuring habitat-related properties, important examples of marine enzymes in biocatalysis will be reported. Completion of this report is devoted to the analysis of novel chemical and stereochemical biodiversity offered by marine biocatalysts with particular emphasis on current or potential applications of these enzymes in chemical and pharmaceutical fields. The analysis of literature cited here and the many published patent applications concerning the use of marine enzymes supports the view that these biocatalysts are just waiting to be discovered, reflecting the importance of the marine environment. The potential of this habitat should be thoroughly explored and possibly the way to access useful biocatalysts should avoid destructive large-scale collections of marine biomass for enzyme production. These two aspects are day by day increasing in interest and a future increase in the use of marine enzymes in biocatalysis should be expected.

## 1. Introduction

Enzyme bioprospecting is a basic research activity devoted to the search for novel biocatalysts. The marine habitat is one of the natural locations of interest for enzyme bioprospecting activity. Sea sources of enzymes are represented by microorganisms or fungi, plants or animals, in particular great efforts are directed towards extremophiles and symbiotic microorganisms. Fishes, prawns, crabs, snakes, plants and algae can represent rich sources of biodiversity, although the most current bioprospecting activity is founded on microbial products. In recent years, enzyme bioprospecting activity has incorporated the new technological system of knowledge (e.g., metagenomics), acquiring new potency and effectiveness especially for enzymes from marine environments.

Remarkable or unusual bioprocesses are performed by marine biocatalysts due to habitat-related characteristics such as salt tolerance, hyperthermostability, barophilicity and cold adaptivity, which can be desirable features recognized from a general biotechnological perspective. The knowledge of enzymes showing optimal activities at different values of salt concentration, pH and temperature is useful, and the comprehension of the interactive effects of these environmental factors is of key importance to exploit an enzyme’s potential. Moreover, a marine enzyme may carry novel chemical and stereochemical properties. Biocatalytically oriented studies (suitable substrates, appropriate reaction conditions, stereochemical asset of catalysis) should be performed to reveal this “chemical biodiversity” which increases interest for these enzymes.

Practical implications in the use of marine catalysts result from a very recent interesting article by Leary *et al.* [1], reporting on the scientific and commercial interest of marine genetic resources. Analyzing the related patents in the period 1973–2007 these authors identified five domains of applications and, among them, chemistry and pharmacology amounted for 53.5 and 32.2%, respectively. Food, cosmetics, agriculture and others were recognized as the remaining industrial domains of interest [1]. Chemical and pharmaceutical fields, covering almost the whole body of applications, strictly rely upon (stereo)chemical characteristics possessed by marine biocatalysts.

A recent review tabulated numerous examples of marine enzymes and discusses their potential for each biochemical class of enzymes. An account of the current knowledge of known examples was presented stimulating future applications [2]. The present review is intended to start from a thorough analysis of habitat-related properties presenting marine enzymes with novel chemical and stereochemical biodiversity.

## 2. Marine Biocatalysts

Marine microorganisms can be found as intracellular or extracellular symbionts, and their hosts are mostly marine animals (vertebrates or invertebrates). These symbiotic microorganisms must possess an arsenal of enzymes and pathways to fulfill the requirements of the host organisms [3].

A number of extremophiles have been isolated from marine environments. Interest for these unique forms of life is based upon their ability to thrive in ecological niches characterized by high or low temperatures, extremes of pH, high salt concentrations and high pressure, and on the robustness of their metabolic machineries [4–6]. Enzymes acting on carbohydrates, proteolytic and lipolytic activities and different alcohol dehydrogenases are known examples identified from these extremophiles. Furthermore numerous extremozymes are discovered by cultivation-dependent and independent approaches [6].

Marine microalgae are also used for biotechnological applications as biocatalysts performing biotransformations of marine natural products or biosynthesis of secondary metabolites and as test organisms in ecotoxicology. Recent interest for marine algae acting as biocatalysts for deep-sea raw material deposition is illustrative [7].

Waste management research concerning fishery and seafood related industries, is a rich source of knowledge about clever and commercially suitable exploitation of marine enzymes from different sources, especially marine animals. Among remarkable examples, digestive juices of abalone and scallops rich in hydrolytic enzymes may be applicable to degrade crystalline cellulose from plant source by-products into glucose [8].

Another field of knowledge greatly concurring to the appreciation of marine sources for enzymes to be used in biocatalysis is the study of marine biomarkers in relation to their significance in pollution monitoring [9]. The general increasing awareness for the potential long-term adverse effects of chemicals and risks for aquatic and terrestrial ecosystems, have greatly enhanced the knowledge in this field; the results regarding enzymatic activities are extremely interesting for biocatalytic exploitation [10–12]. As an example, it is highly significant that developments in this field lead to the identification of proteomic signatures of exposure to marine pollutants in mussels (*Mytilus edulis*). A unique set of protein expression (signature) for exposure to different chemical compounds has been recognized and the expressed proteins were identified to participate in α- and β-oxidation pathways, in xenobiotic and amino acid metabolism, in cell signaling, and in oxyradical metabolism [13].

Another very interesting case study is the collection of information aimed to discover the potential of bivalve mollusks (or microorganisms associated to them) to catabolize marine biotoxins, especially the paralytic shellfish toxins for which commercial detoxification methods are difficult or do not exist. An algal extract containing a mixture of different toxins (saxitoxin, neo-saxitoxin, gonyautoxins and others) was used for the analysis, and after five days of incubation the reduction of overall toxicity was verified by mouse bioassays and HPLC. Novel strains of *Pseudoalteromonas haloplanktis* were identified capable of catabolizing the biotoxins analyzed [14]. Enzymes involved in such reactions could be of extreme interest in semi-synthetic steps adopted for biotransformation of bioactive natural products.

Generally metagenomic strategies hold great potential to study and exploit the enormous microbial biodiversity present within marine environments. The powerful cultivation-independent approach of metagenomics can be applied today to gain access to biocatalysts from uncultured marine microorganisms [15–19]. The information on genome sequence and the possibility to express clone libraries for all ORFs have made possible the use of recombinant proteins to try to associate them to specific activities.

Another very powerful tool for discovering novel enzymes of possible biotechnological interest is the identification and study of marine microorganisms with unique physiological traits. This approach can complement the enormous amount of data concerning gene diversity in marine environments offered by metagenomics. A case study is the Gram-negative marine bacterium *Marinomonas mediterranea.* First selected because of unusually high amounts of melanins, its study has allowed the cloning of several genes encoding oxidases of biotechnological interest [20].

Studies on the production of marine biocatalysts based on different bioprocess engineering approaches are interesting. Using three major modes of operation: batch, fed-batch and continuous, laboratory and pilot plant scale bioreactors of this type for protease, chitinase, agarase, and peroxidase production from marine bacteria or fungi were recently described in a comprehensive overview on the various bioprocess strategies adopted for the cultivation of marine organisms for production of enzymes [21].

In the context of bioprospecting marine biocatalysts, along with several high-throughput screening methods to facilitate the identification of the best candidate biocatalyst, a metabolomic approach for enzyme-function discovery is a very interesting complementary technique that can be profitably cited here [22]. In practice, incubation of a mixture of metabolites with the expressed enzyme and analysis by mass spectrometry of the reaction mixture allows identification of specific changes in the metabolite composition directly suggesting substrate(s) and product(s) of the reaction and the presence of an enzymatic activity.

## 3. Habitat-Related Properties of Marine Biocatalysts

As introduced above, the interest in marine enzymes is related to the ecological features of habitat in which marine organisms thrive which impact on their metabolic functions enabling their (bio)molecular machinery. The resulting enzymatic properties are very important from a biotechnological point of view (e.g., extremophiles). In this section, an analysis of these habitat-related distinctive features is presented, reporting important biocatalysis-related examples.

### 3.1. Salt and pH Tolerance

Proper recognition of the industrial potential of halophilic enzymes is increasing and various benefits are attractive. The synthesis and production of polyhydroxyalkanoates by halophiles is one of the current biotechnological topics related to these organisms [23]. However, highly interesting in our context is the strict relation among salt and organic solvent tolerances very often observed for halophilic enzymes because salt presence has the effect of reducing water activity [24].

Interestingly, in the range of sodium chloride concentration below the value characterizing moderate halophiles and more common in marine organisms, enhancement of thermostability can be also observed [5]. Salinity of seawater is about 3.5% (≥0.6 M NaCl) while halophilic microorganisms are considered moderate if they can grow at salt concentrations between 0.85 and 1.7 M NaCl and as extreme halophiles if they require NaCl concentrations above 1.7 M for growth [24]. Thermal stability studies have been performed on numerous proteins from several hyperthermophiles and glutamate dehydrogenase (GDH) is one of the most thoroughly studied enzymes in this domain used as a model for understanding the molecular mechanisms involved in thermostability. The marine microorganism, *Pyrococcus furiosus*, was shown to possess a glutamate dehydrogenase showing substantially enhanced activity as well as thermostability at increasing salt concentrations. The results of a comparative analysis of enzyme from freshwater and marine hyperthermophilic archaea [25] pointed to the existence of different strategies for achieving thermostability between marine and freshwater species. In marine species, the high availability of salts might contribute to stabilizing interactions by participating in electrostatic attractions within the protein complex.

Marine amylases are of interest from a biotechnological perspective. The microbial production of ethanol from carbohydrates is mainly dependent on the process of saccharification requiring enzymes to hydrolyze carbohydrates before fermentation. Biocatalysts used are mainly terrestrial amylases, hence the bioprocesses of ethanol production using marine microalgae biomasses must include desalinization if a terrestrial enzyme is to be used, thus the development of a system utilizing amylase from a marine source would be very beneficial. An amylase-producing marine bacterium, *Pseudoalteromonas undina* NKMB 0074, was isolated and identified: it can be used in saccharification of marine microalgae producing ethanol, in saline conditions [26]. A novel α-amylase from marine *Streptomyces* sp. D1 has also been very recently reported. Interestingly, in this case the hydrolysis pattern indicated that it has both α-1–4, α-1–6 (debranching) activity [27]. Activity of this enzyme drastically decreased in the absence of NaCl after 48 h incubation; the enzyme retained almost 100% and 50% of its activity in the presence of 7% (w/v) NaCl and 10% (w/v) NaCl after 48 h incubation. The amylase enzyme was found to be stable in phosphate buffer systems having pH 7–8 as well as glycine-NaOH buffers in the pH range 9–11 when incubated for 6–48 h. This is the first report for a *Streptomyces* enzyme having such a wide range of pH stability, and therefore potential widespread application in the detergent industry is conceivable. This strain showed good growth in the temperature range of 37–55 °C but optimum growth and maximum amylase production were observed at 45 °C. The enzyme retained almost 50% of its activity at 85 °C. This clearly indicates the moderately thermostable nature of enzyme. Until today, bacteria belonging to the genus *Bacillus* have been exploited for commercial production of thermostable amylase enzymes but there is no such report on isolation of moderately thermostable amylase enzymes from marine *Streptomyces*.

In a context of study about proteases successfully used for the production of biologically active fish protein hydrolysates, the enzyme trypsin from the intestine of carnivorous fish smooth-hound (*Mustelus mustelus*) showed high proteolytic activity at very high (30%) NaCl concentration, demonstrating its potential for protein hydrolysis at high salt content [28], and confirming its interest as a possible biotechnological tool in the fish processing and food industries.

One of the strategies for osmoregulation is based on the synthesis and/or accumulation of compatible solutes not interfering with enzymatic activity. A variety of such molecules are known, including glycine betaine, ectoine, trimethylamine oxide, urea and other amino acid derivatives as well as sugars and sugar alcohols. d-Alanine, found in specialized peptides and antibiotics, and present also as a component of peptidoglycans, is involved in the response to osmotic stress in several marine invertebrates. Indeed, alanine racemase activity was detected in the homogenates of the tissues of these organisms. This enzyme has been purified to homogeneity from the hepatopancreas of the black tiger prawn, *Panaeus mondon* [29]. The prawn enzyme is activated and stabilized by the presence of monovalent anions including chloride. This is consistent with the previous hypothesis that d-alanine serves as an osmoregulator in marine animals [29]. Unfortunately the enzyme acts almost exclusively toward alanine but interestingly l-serine, which is racemized at a rate of about 0.5% of that of l-alanine, is the only exception.

### 3.2. Hyperthermostability

A biotechnological process performed at elevated temperature possesses many advantages: high solubility of substrates, in particular for poorly soluble or polymeric molecules, decreased viscosity of reaction mixtures, faster reaction rates and increased (bio)availability of substrates, decreased risk of microbial contamination. Conventional proteins are completely denatured under the harsh conditions in which hyperthermophilic enzymes can operate.

Both terrestrial and marine microorganisms may be adapted to grow optimally under these conditions (80–108 °C). Enzymes derived from extremophilic archaea have higher stability towards heat, pressure, detergents, solvents and they are often more resistant to proteolytic attack [30]. Research has shown that there are a variety of mechanisms possible to impart both thermostability and interesting chemical and stereochemical features to these enzymes.

Hyperthermophilic biocatalysts for oxidation-reduction reactions with interesting properties are mentioned in a recent general reference on dehydrogenases [31]. Ketoreductases possessing anti-Prelog enantioselectivity, or broader substrate specificity, with larger active site for bulky substrates and higher thermostability and tolerance to organic solvents, are particularly required. Biocatalytic characteristics such as substrate specificity and enantioselectivity of an alcohol dehydrogenase from the marine hyperthermophilic archaeon *Pyrococcus furiosus* have been fully evaluated for major guidance in future biocatalytic applications. This enzyme catalyzes the reduction of various ketones including alkyl and aryl ketones, and α- and β-ketoesters. Aryl ketones were reduced to the corresponding chiral alcohols in an enantiomerically pure form. Interestingly the reaction temperature increased the enzyme activity, but exerted no decreasing effect on the enantioselectivity. This alcohol dehydrogenase showed also an attractive high tolerance towards organic solvents such as dimethyl sulfoxide, iso-propanol, methyl tert-butyl ether, and hexane, a useful feature for working with ketones possessing low solubility in aqueous buffers [32].

Thermophilic marine microorganisms belonging to *Thermotoga* sp. possess interesting glycoside hydrolases often used in transglycosylation reactions [33–35]. The β-glucosidase from the hyperthermophilic eubacterium *T. maritima* has been expressed to high levels in *E. coli*, and purified to homogeneity by heat precipitation and ion exchange chromatographies. The enzyme is extremely thermophilic, thermostable and resistant to common protein denaturants as are other enzymes from the *Thermotoga* species and the activity is also stimulated in the presence of alcohols and organic compounds. A series of straight chain aliphatic alcohols were adopted as acceptors of sugars in transglycosylation in the presence of 15% DMSO demonstrating that as the chain length of the alcohol increased, the rate of release of *p*-nitrophenol also increased. Maximal activity was induced by the presence of hexanol while higher carbon chain alcohols reduced the rate of activation. These properties make this β-glucosidase a good candidate as an enzyme for use in industrial applications such as alkyl glucosides production [33]. A recent paper describes a series of enzymatic transglycosylation reactions performed using the crude homogenate of marine *Thermotoga neapolitana*. The study was focused on synthetic features of several transglycosylating enzymes present in the homogenate. Xylosidase/xylanase activity, the most abundant, led to convenient syntheses of interesting series of pure (β-1,4)-xylooligosaccharides of different aglycones starting from xylan as donor and different acceptors such as 1-hexanol (producing promising candidates for new surfactants), 9-fluorene methanol (obtaining anti-HSV agents), 1,4-butanediol (for the synthesis of new glycolipids), and geraniol (producing aroma compounds). Furthermore, the regioselectivity during galactose, fucose, glucose, and mannose enzymatic transfers was also investigated [35]. Composition of the reaction mixture using hexanol as an acceptor indicated that up to xylotetraosides were present as products; however hexyl β-xyloside represented the kinetically favored product obtained in a final concentration of 19.5 mM at 48 h, corresponding to a molar yield of *ca.* 17% with respect to xylose available (average molecular weight of xylan 25.000). This high yield, corresponding to a concentration of *ca.* 5 g/L (228 mg/g of xylan) is an order of magnitude higher than that obtained using *Trichoderma longibrachiatum* xylanase and four-fold higher than that obtained using *Aureobasidium pullulans* in the synthesis of octyl β-xyloside, a very similar compound. Good yield of monoxyloside was also obtained using the bifunctional 1,4-butanediol as acceptor, useful as synthon in the synthesis of new glycolipids. High concentrations of organics (miscible solvent and acceptor itself) used in the reaction mixtures for the synthesis of xylooligosides by *T. neapolitana* xylanase are permitted by the unusual resistance of the thermophilic enzyme(s).

Very recently Verenium Corporation developed Fuelzyme^®^ originating from a deep sea bioprospecting research, possessing α-amylase activity for mash liquefaction. The enzyme is currently marketed claiming broad temperature and pH operating ranges allowing greater operating flexibility [36]. It has a unique mode of action randomly hydrolyzing internal α-1,4-glucosidic bonds in starch and its degradation products thus liberating a consistent blend of lower molecular weight, soluble dextrins and oligosaccharides. This biocatalyst can be used for the liquefaction of barley, corn, potato, rice, sorghum, wheat, and other starch-based mashes and slurries.

### 3.3. Barophilicity

Interest for the pressure effects on biochemical processes is well documented [37]. The behavior of a system under high pressure is governed by Le Châtelier’s principle: application of pressure shifts an equilibrium towards the state with smaller volume, thus favoring processes for which the transition state has a smaller volume than the ground state. Undoubtedly the basic advantage of pressure as enzymatic reaction controller is based on the fact that no new chemical agents are added in the reaction mixture.

Depth of the oceans is estimated on average to be *ca.* 4000 m; consequently, life under the oceans must be able to withstand considerable pressure. Indeed vertebrate fish have been observed at depths of *ca.* 10,000 m in the deepest ocean sites, while metabolism in piezophiles (barophiles) has been shown to be viable at artificial pressures of an order of magnitude higher. In addition to living at high pressures, piezophilic organisms are typically psychrophilic or thermophilic in nature due to the cold temperatures of the deep ocean or to the proximity to hydrothermal vents.

A wide range of biotechnology related areas such as deep-sea waste disposal, production of novel natural products and catabolic activities, and in general the provision of enzymes for high-pressure bioreactors, all benefit from the study of pressure effects on biochemical processes. Attention has been directed also to the potential biotechnological applications of piezophiles compared with those of other extremophiles [38].

Research on barophiles has focused mostly on the identification of pressure-regulated operons showing the relationship between pressure, high temperatures and microbial growth [39]. Molecular views for the pressure adaptation are still subject of debate. Very recently, Hay *et al.* [40] concluded that there was no evidence of pressure adaptation in either the structure or the activity of dihydrofolate reductase from the marine psychropiezophilic bacterium *Moritella profunda*, isolated from great depth. Canganella *et al.* [41] observed that at high pressure (60 MPa) and temperature (90 °C), the number of protein bands remained unchanged when the hyperthermophilic archaeon *Thermococcus peptonophilus* was grown under high pressure and temperature but cell growth was accompanied by the overproduction of specific proteins. This organism was isolated from a hydrothermal vent area at a depth of 1400 m. Of particular interest was the mention of a unique method for the isolation of barophiles from intestinal contents of deep-sea fishes [42].

The function of biological membranes and the dynamic state of lipid components are closely related. The fluidity of a membrane at 100 MPa and 2 °C (typical deep-sea conditions) is similar to that at atmospheric pressure and −18 °C. Increasing pressure, like a reduction in temperature, tends to solidify phospholipids going from a liquid-crystalline state to the gel state disrupting the function of the cell membrane. There is a striking correlation between growth at high pressure and fatty acid unsaturation index. An increase in unsaturated fatty acids leads to highly disordered phospholipid bilayers rendering the membrane workable against pressure effects. In this context, piezophilic bacterial lipid biochemistry is a very interesting aspect and enzymes acting in these metabolic routes under these extreme conditions could be very interesting for biocatalytic applications [43].

Pressure alters also the structure of the cell wall and cytoskeleton. The up-regulation by pressure of small hydrophilic proteins acts by improving the flexibility of the cell wall disrupting interactions between adjacent polysaccharide layers that would otherwise build a rigid structure [44]. Also in these cases, enzymatic machinery is of interest for biocatalytic applications related to supramolecular chemistry of polysaccharides.

### 3.4. Cold Adaptivity

The considerable potential of enzymes acting at low temperature for biotechnological exploitation is known: these biocatalysts could be utilized as additives in the detergent and food industries, or in bioremediation processes, minimizing energy consumption, reducing the risk of microbial contamination, avoiding the high temperature instability of reactants or products. The use of psychrophilic enzymes can be advantageous not only for their high specific activity (allowing reduction of the amount of enzyme needed) but also for their easy inactivation preventing prolonged action of the enzymes when undesired.

Isolation and characterization of bacteria that are able to efficiently remove lipids at low temperatures will provide insight into the possibility to use cold-adapted microorganisms as a source of exploitable enzymes. The lipolytic activity of cold-adapted antarctic marine bacteria was studied [45]. The enzyme activities were rather variable and occurred at a wide range of salt concentrations and pH’s in accordance to the bacterial physiology. These isolates could be interesting sources of enzymes to be exploited in specific industrial processes such as bioremediation of fat-contaminated aqueous systems.

Generally the thermostability is associated with a pronounced rigidity of the molecular structure and this impairs the substrate-enzyme interactions, leading to a weak specific activity. On the contrary, flexibility or plasticity characterizing cold-adapted enzymes has as a consequence high specific activity. This inherent greater flexibility will be particularly useful in conditions wherein the activity of mesophilic and thermophilic enzymes is severely impaired by an excess of rigidity [46]. When the water associated to the protein falls below a certain level, the enzymes become more rigid and then perhaps less efficient. Psychrophilic enzymes may have a potential advantage also for applications under low water conditions with the consequence of increasing activity.

The psychrotolerant bacterium *Shewanella* sp. G5, isolated from the intestinal content of *Munida subrrugosa*, can actively use cellobiose as carbon source. The presence and characterization of inducible cold-active β-glucosidases is reported [47]. Although not yet studied, selectivity in both hydrolysis and transglycosylation reactions typical of these enzymes could be of interest.

So far, only a few cold-active esterases and lipases from psychrophilic microorganisms have been cloned and characterized. A recent study was focused on a cold-adapted esterase of a novel marine isolate, *Pseudoalteromonas arctica*. In particular, gene cloning, enzyme purification and characterization were reported. Among properties of interest, the esterase displays broad substrate specificity for short-chain fatty acid esters (C2–C8) and shows an optimum pH of 7.5 and optimum temperature of 25 °C, but with more than 50% retained activity at the freezing point of water. Furthermore, the enzyme was capable of hydrolyzing esters of medical relevance such as non-steroidal anti-inflammatory drugs (naproxen, ketoprofen, and ibuprofen) [48].

### 3.5. Novel Chemical and Stereochemical Properties

Novel chemical and stereochemical properties can be found among marine biocatalysts acting as a booster of interest in marine sources from a biocatalytic point of view. Substrate specificity and affinity are evolved properties related to the metabolic functions of the enzymes and to the ecologies of the marine sources from which they were derived.

Important examples have been found in the class of oxidoreductases, of carbohydrate active enzymes and others. For the oxidoreductases, different stereoselectivities could be observed with respect to their terrestrial counterparts adding flexibility with regard to tolerance of high salt concentrations and/or resistance to organic solvents (see alcohol dehydrogenase from the hyperthermophilic archaeon *Pyrococcus furiosus* mentioned above).

**Figure 1.**
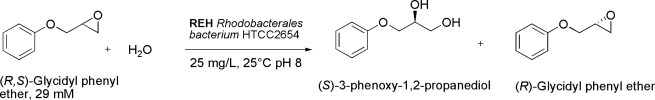
Reaction of REH (*Rhodobacterales* epoxide hydrolase) with racemic glycidyl phenyl ether forming pure (*R*)-epoxide (*e.e.* 99.9%) in 38.4% yield.

Marine epoxide hydrolases may have enantioconvergent applications for the production of interesting diols with specific stereochemistry [49–52]. Epoxides and derived diols in pure stereochemical forms are synthetic intermediates for drug production, thus great efforts in the study of catalysis by epoxide hydrolases have been carried out. Adopting the so-called enantioconvergent process, two different enzymes can be used each possessing different specificity for the attachment to the α- or β- carbons of an epoxide ring. In this method, one enzyme acts on a specific carbon while the complementary biocatalyst hits the opposite, resulting in theoretical yield of 100% diol with specific configuration. Thereby, sources of different epoxide hydrolases, to be used in combination, are of extreme interest for enantioconvergent applications. A recent success in the synthesis of (*R*)-phenyl-1,2-ethanediol from racemic styrene oxide involves the use of an epoxide hydrolase from the marine fish *Mugil cephalus* [49]. Enantioselective epoxide hydrolases activities were also found in the microorganism *Sphingomonas echinoides* isolated from seawater [50] and from another fish *Danio rerio* [51]. Enantiopure (*S*)-styrene oxide was prepared from its racemate, with a yield of 21% from an initial concentration of 40 mM substrate using the whole-cells of *S. echinoides*. (*S*)-styrene oxide with an enantiomeric excess (*e.e.*) higher than 99% was readily obtained also using the recombinant fish EH of *D. rerio* [51]. Enantioselective resolution of a wide range of racemic epoxides and the molecular basis of EH gene are under investigation in more detail [50]. Highly enantioselective epoxide hydrolase was recently found in the marine bacterium *Rhodobacterales bacterium* HTCC2654. Racemic glycidyl phenyl ether (Figure 1) formed enantiopure (*R*)-GPE from kinetic resolution of 29.2 mM racemic substrate in yield of 38.4% (theoretical, 50%). Authors claimed that this study demonstrates the highest enantioselective resolution of racemic glycidyl phenyl ether (E-value of 38.4, was calculated with an *e.e.* of 99%) using a purified biocatalyst among the known native epoxide hydrolases [52].

**Figure 2.**
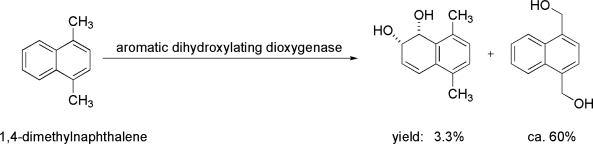
Reaction of transformation of 1,4-dimethylnaphthalene using the cells of *Escherichia coli* expressing aromatic dihydroxylating dioxygenase.

Non-activated carbon atoms are difficult to manage by pure chemical routes. In the realm of enzymes used by marine organisms to face environmental pollution, very interesting examples of these biocatalysts can be noticed, all characterized by a potent chemical action on inert structural positions. Also, in these cases, substrate preferences and positions of functionalization of marine enzymes differ from terrestrial examples. Polycyclic aromatic hydrocarbons (PAHs) are a class of organic pollutants present in the marine environment. They are composed of two or more fused aromatic rings. Persistence, ability to bioaccumulate and carcinogenicity of PAHs are all important aspects of interest concerning the need for biotransformation of these compounds [53]. Results of experiments using various mono- or di-substituted naphthalenes such as dimethylnaphthalenes using the cells of *Escherichia coli* expressing aromatic dihydroxylating dioxygenase genes of marine bacteria, *Nocardioides* sp. KP7 and *Cycloclasticus* sp. A5, respectively, were reported. The authors shed light about the broad substrate preference of enzymes which were often able to hydroxylate methyl groups. Specifically, 1,4-dimethylnaphthalene was predominantly bioconverted into 1,4-dihydroxymethylnaphthalene [54], which possess applications as starting materials for the synthesis of industrially useful chemicals (Figure 2). The diversity and distribution of alkane hydroxylase genes in sediments of the Timor Sea was also studied. Protein sequences derived from clone libraries suggest that the Timor Sea may be a rich reservoir for novel alkane hydroxylase enzymes [55]. In the list of microorganisms representing a potentially valuable system for replacing chemical oxygenating processes of the normal alkanes (n-alkanes), *Alcanivorax borkumensis* has a unique position as it is a key bacterium involved in the biodegradation of oil spills from marine environments. Recently Miri *et al.* demonstrated that the alkane hydroxylase AlkB2 from *A. borkumensis* has potential as a biocatalyst in the biotransformation reactions of fatty acids as well as alkanes [56].

A large number of enzymes possessing particular catalytic characteristics can be outlined also for marine biocatalysts acting on carbohydrates. Among others, the tendency of polyglycosylation is an interesting common quality of *Aplysia* α-glucosidase and other enzymes of marine origin during transglycosylation reactions. *Aplysia* is a genus of sea hares belonging to the family *Aplysiidae*, containing different species of organisms. *Aplysia fasciata* Poiret, 1789 which is one of them, is very common in Mediterranean habitats. The *Aplysiidae* are herbivorous, eating a variety of red, green or brown algae. They are potent producers of a library of glycoside hydrolases applied in the synthesis of glycosidic bonds [57]. A β-mannosidase with catalytic efficiency significantly higher than those reported for β-mannosidases from other sources has been also reported in this marine organism [58]. It possesses exo-acting activity and when the enzyme is incubated in the presence of *p*-nitrophenyl β-d-mannopyranoside, self-transfer of the mannosyl group is observed with formation of 10–15% yield of β-1–4 disaccharide. In the presence of a heteroacceptor such as *o*-nitrophenyl α-d-2-deoxy-*N*-acetyl glucopyranoside, two regioisomers (85:15, 12% yield) due to the β-mannosylation in 4 and in 6 positions were formed. In the same organism an α-glucosidase and a β-galactosidase were also detected, purified and used for different transglycosylation reactions using various acceptors [59,60]. As far as the β-galactosidase is concerned, results of transgalactosylation reactions indicate a clear preference of the *Aplysia* enzyme for the galactosylation of polar acceptors. Owing to the specificity of the acceptor site of most terrestrial galactosidases for apolar compounds with phenyl groups, the yields obtained in the reactions using free or methyl derivative of xylose and methyl β-galactopyranoside and D-galactose, are interestingly high for this marine enzyme. Another exciting characteristic of this enzyme from *Aplysia* is the uncommon β-1,3 selectivity in the transgalactosylation reactions with most of the acceptors. Enzyme regioselectivity was absolute in reactions for the synthesis of nucleoside derivatives. In these cases only the product of galactosylation at the 5′ position of the nucleosides was observed. Reaction yields were satisfactory in most cases, and very high for uridine derivatives. In particular, 5′-*O*-β-galactosyl-5-fluorouridine, the galactosylated derivative of the anticancer drug fluorouridine, was synthesized with a 60% yield, and 5′-*O*-β-galactosyl-3′-azido-3′-deoxythymidine, the derivative of the anti-HIV drug, was obtained in 43% yield [61]. This was the first report dealing with a glycoside hydrolase used for the modification of nucleosides with such high yields.

**Figure 3.**
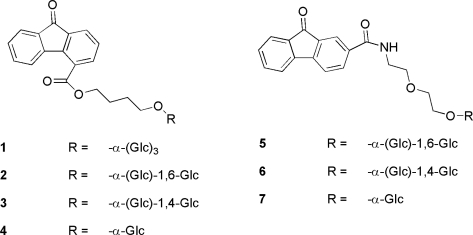
Products formed by glucosylation of 9-fluorenone carbohydroxyesters (**1**–**4**) and carbohydroxyamides (**5**–**7**).

Characterization of performances of the α-glucosidase from *Aplysia* in transglycosylation reactions indicated the preferential enzymatic formation of α-1–4 linkages in the early stages of reaction and the accumulation of α-1–6 products which were confirmed by time course experiments using maltose as donor and acceptor [59]. The reactions in which cellobiose, saccharose, pyridoxine, naringin and 9-fluorenone derivatives were used as acceptors and maltose as donor, were studied in detail. High-yielding enzymatic α-glycosylation of pyridoxine using this marine enzyme were obtained after optimization of reaction conditions, reaching *ca.* 80% molar yield of products (pyridoxine monoglucosides 24 g/L; pyridoxine isomaltoside 35 g/L). High selectivity toward the 5′ position, in both mono- and disaccharidic material, was observed along with traces of trisaccharidic derivatives of pyridoxine [62]. The enzymatic glucosylation of naringin has been also possible. The regioselective formation of both the mono-α-glucosyl derivative at β-gluco-C6 position of the natural product, and of the corresponding isomaltosyl diglucoside of naringin is observed in high yield and efficiency of reaction: suspensions of insoluble naringin can be used up to *ca.* 90 mg/mL initial acceptor concentration. Interestingly, in different experiments it was demonstrated that one of diasteromers of the naringin is preferred by the enzyme from *A. fasciata* during glucosylation/deglucosylation enzymatic steps. Finally, the feasibility of efficient naringin glucosylation directly within grapefruit juice is also demonstrated at low maltose concentrations and optimal pH of the enzyme [63]. Other acceptors belonging to 9-fluorenone derivatives (Figure 3) were used providing several α-*O*-polyglucosides which were useful for a quick screening of the pharmaceutical profile as modified by carbohydrate(s) moieties with respect to parental antiviral aglycones [64].

The tendency for polyglycosylation of the α-glucosidase from *Aplysia* in transglycosylation reactions observed in the experiments reported above, is a useful asset of the enzyme from a biocatalytic point of view as it allows easy access to a series of glycosides and is a characteristic common in other enzymes of marine origin (see also below). An α-glucosidase from *Geobacillus* sp., one of the deepest sea bacteria isolated from the sediment of the Mariana Trench, has been reported. It is a thermo- and alkaline-stable enzyme exclusively hydrolyzing α-1,4-glycosidic linkages of oligosaccharides in an exo-type manner. The enzyme presented an overwhelming transglycosylation activity and glycosylated various non-sugar molecules such as alkyl alcohols, chloramphenicol, cortisone, and phenolphthalein when maltose was used as a sugar donor. Usually a three-product pattern of polyglycosylated compounds was observed by TLC although details about interglycosidic linkages formed were not reported [65].

In another example of polyglycosylation enzymes the nature of the monosaccharide is different. l-fucose is one of the most common monosaccharides at the nonreducing end of many glycans and it is indeed an important biological determinant. Its terminal location on glycoconjugates makes it sensitive to the action of α-l-fucosidases which are thus involved in many important biochemical processes such as plant defense, inflammation, metastasis and in the genetic disease named fucosidosis. An interesting α-l-fucosidase has been identified in the digestive glands of the common marine mollusk *Pecten maximus* [66]. The authors appreciated the high catalytic activity compared with other known fucosidases and their report seems to be the first describing an enzymatic activity of fucosidase type that can efficiently release fucose from fucoidan (exo-acting activity) [66]. This enzyme has an interesting transfucosylase activity and is able to synthesize fuco-oligosaccharides higher than disaccharides by transglycosylation processes. The produced disaccharides, despite their low concentrations, can undergo further transfucosylation events even in the presence of high concentrations of the acceptor, leading to the formation of trisaccharides and in turn of tetrasaccharides. All new glycosidic linkages are of α-l type and highly branched products are formed [67].

**Figure 4.**
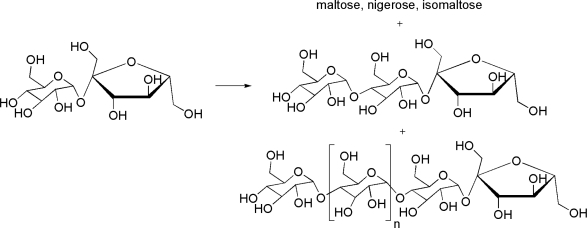
Products of sucrose transglycosylation by purified α-glucosidase from *A. ventricosa*. Polyglucosylfructoses were produced with DP3 to DP8.

As another case of polyglucosylation by marine enzymes, sucrose transglycosylation was studied. The digestive juice in the stomach of the snail *A. ventricosa*, an edible gasteropod mollusk of humid sub-Saharian Africa, is the source of a novel α-glucosidase active on sucrose, pNP-α-d-glucopyranoside and maltose [68]. The authors investigated the transglycosylation potential of this enzyme using sucrose as single glucosyl donor and acceptor substrate in order to access specific features of interest in polyglucosylfructosides synthesis. The products of this transglycosylation reaction were purified and their structures were characterized by HPAEC-PAD, LSI-MS, enzymatic hydrolysis and NMR spectroscopy establishing a 3–8 degree of polymerization. The mixture of mono-, disaccharides and polyglucosylfructosides obtained from sucrose (Figure 4), can be used in a series of food applications such as acariogenic sweeteners, to impart body to food products or to prevent sugar crystallization.

In this general important domain of enzymes for carbohydrate manipulation, a new endo-type β-agarase can be mentioned. It was obtained from a newly isolated marine bacterium *Agarivorans* sp. LQ48. The gene expressed in *E. coli* produced a mature agarase hydrolyzing the β-1,4-glycosidic linkages of agarose units, yielding neoagarotetraose and neoagarohexaose as the main products. These products from agar or agarose, possess diverse biological functions and have many potential applications in the food, cosmetic, and medical industries. It is remarkable that this enzyme still retained more than 95% activity after incubation at pH 3.0–11.0 for 1 h, a characteristic much different from other agarases previously reported [69].

Mannan and related degradation products, *i.e.*, mannooligosaccharides, have been attracting research attention in the food and pharmaceutical industries for various beneficial effects on human health that these molecules possess. A mannan-degrading activity has been recognized as an important enzyme to produce the above bioactive mannooligosaccharides. This enzyme was isolated from the digestive fluid of the common sea hare *Aplysia kurodai*. The purified protein could degrade glucomannan and galactomannan as well as the linear β-1,4-mannan, indicating that it is a typical endo-β-1,4-mannanase splitting internal β-1,4-mannosyl linkages of mannan [70].

The hyperthermophile isolated from marine environments, *Thermotoga maritima*, is slightly halophilic (optimum NaCl concentration of 2.7%, wt/vol) and adopts biochemical strategies to counterbalance the external osmotic pressure. The organic solutes discovered in organisms living in hot environments are different from those used by mesophiles, leading to the view that osmolytes of (hyper)thermophiles could play an additional role as protectors of macromolecules and other cellular components against heat damage. Thus the enzymatic machinery for biosynthesis of these compounds is of extreme interest. In addition to di-myo-inositol-1,3-phosphate (DIP), the organic solute pool of *Thermotoga maritima* comprises two mannosylatyed derivatives 2-(*O*-β-d-mannosyl)-di-myo-inositol-1,3-phosphate (MDIP) and 2-(*O*-β-d-mannosyl-1,2-*O*-β-d-mannosyl)-di-myo-inositol-1,3-phosphate (MMDIP). The synthesis of MDIP involved the transfer of the mannosyl group from GDP-mannose to DIP in a single-step reaction catalyzed by MDIP synthase. This glycosyltransferase can use MDIP as an acceptor of a second mannose residue, yielding the di-mannosylated compound. Tri-mannosylated form was also detected in minor amounts. The stereochemistry of MDIP synthase was determined by NMR and stable isotopic labeling. This 1,2-mannosyltransferase is unrelated to known glycosyltransferases, and, within the domain of Bacteria, it is restricted only to members of the two deepest lineages, *i.e.*, the *Thermotogales* and the *Aquificales* [71].

**Figure 5.**
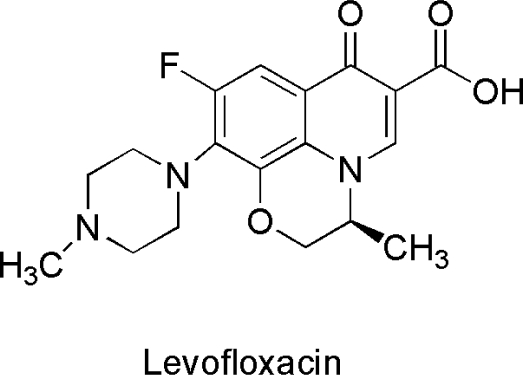
The *S*-isomer of the antibiotic ofloxacin called Levofloxacin obtained by enantioselective resolution of racemic ofloxacin propyl ester using the novel esterase from *Yarrowia lipolytica* CL180.

Hydrolytic enzymes acting on lipid molecules (esterases, lipases, *etc.*) constitute a wide class of biocatalysts known for a long time and fruitfully adopted in solving many problems in organic synthesis (enantioselective hydrolysis and acylation reactions) at both lab scale and industrial processes. Lipases have emerged as key enzymes in food, chemical, pharmaceutical, cosmetic and detergent productions, leather processing, and biodiesel production since many years. As far as marine lipid active hydrolases are concerned, interesting unusual characteristics could be found. The case of the novel esterase from *Yarrowia lipolytica* CL180 for the enantioselective resolution of racemic ofloxacin propyl ester is very important and deserves mention, in this respect [72]. Levofloxacin, the *S*-isomer of ofloxacin (Figure 5), shows broad spectrum antibacterial activity against both gram-positive and gram-negative bacteria and the activity is doubled with respect to racemic ofloxacin. This recent study presents screening of marine organisms, from a variety of marine environments such as cold sea, hydrothermal vent area, sediment, tidal flat area, arctic sea. Cloning, overexpression, and biochemical characterization of the novel esterase from *Yarrowia lipolytica* CL180 was possible and the enantioselective resolution of racemic ofloxacin propyl ester using the recombinant enzyme was set up. The esterase exhibited psychrophilic activity and, in fact, still showed 40% of the maximal activity at 10 °C. These features make it a very attractive enzyme for potential application in producing a heat-labile chemical although the enantiomeric excess of 52.1% obtained in the example reported should be improved.

In the realm of hydrolytic enzymes, marine proteases are also important. Cyclic peptides are natural products known from marine ascidians, sponges and different genera of cyanobacteria. The biosynthesis of these diverse peptides, called cyanobactins, has been intensively studied and a recent report about proteins responsible for cyclization appeared. Two proteases have been recognized for cyclization process and no addition of energy (ATP) is required. The spectacular example of PatG, one of the two enzymes serving as a useful biocatalyst for the cyclization, is remarkable. It is highly tolerant of diverse substrate sequences and more than 30 natural peptide sequences appeared to be cleaved and cyclized by this enzyme. This study is very important for the possibility that PatG could serve as a useful general biocatalyst for the cyclization of peptides [73].

**Figure 6.**
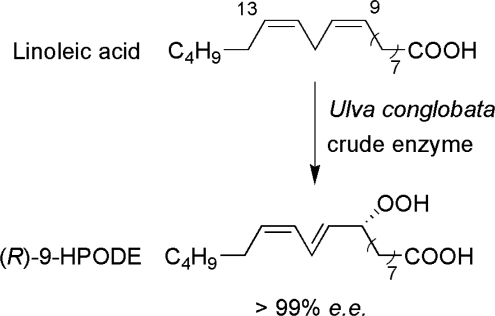
*Ulva conglobata* crude enzyme reaction forming (*R*)-9-hydroperoxy-(10*E*,12*Z*)-10,12-octadecadienoic acid ((*R*)-9-HPODE).

Numerous marine oxidoreductases are reported in literature [2]. An essential oil, which could be prepared by distillation of marine alga *Ulva conglobata*, contains different long- and short-chain unsaturated aldehydes which are formed from long-chain unsaturated fatty acids such as linoleic (LA) and linolenic acid (LNA). Lipoxygenases (EC 1.13.11.12, LOX) catalyze the oxygenation of fatty acids containing a (1*Z*,4*Z*)-pentadiene moiety in a regio- and stereoselective manner involving the corresponding hydroperoxides converted into aldehydes. It has been reported that when LA and LNA were incubated with a crude enzyme of *U. conglobata*, the corresponding hydroperoxides (*R*)-9-HPODE and (*R*)-9-HPOTrE were formed (Figure 6) with a high *e.e.* (>99%), respectively. This regio- and stereoselective way differs from those found in plants and in other organisms and this is of interest in the applicative aspect of lipooxygenases [74]. From the synthetic chemist’s point of view, the asymmetric oxygenation reaction of unnatural substrates has remarkable potential.

Nitrile manipulating enzymes are of great interest in biocatalysis for production of specialty chemicals or in pharmaceutical industry. A comparative study concerning nitrile modifying activities in deep-sea and terrestrial actinomycetes is present in literature [75]. Using the metabolic profiling technique and activity assays it was confirmed that all strains catalyzed the hydrolysis of nitriles by a nitrile hydratase/amidase system. However, authors reported that most of the deep-sea strains had constitutive activities and showed some of the highest activities and broadest substrate specificities of organisms included in their study.

A general updated review examining what is known about pathways of catabolism of steroids in marine and freshwater fish has been recently published [76]. It seems important to mention it here since hydroxylation, glucuronidation and sulfonation of hydroxyl group, are the principal reactions greatly affecting biological activity of steroids. Glucuronides have considerably lower potency than the unconjugated steroids but they are not biologically inert; androgen glucuronides excreted in urine of males attract female fish by acting as pheromones. Formation of sulfate esters of steroids is a major pathway of steroid catabolism in fish and reduces binding to estrogen or androgen receptors; however they still exhibit biological activity: in mammals, sulfated steroids have been shown to interact also with the olfactory system.

## 4. Conclusions

In the present review, the potential of marine enzymes as useful tools in biocatalysis is presented. Starting from the features of the habitat in which marine organisms thrive, the biochemical enzymatic characteristics have been pointed out as important aspects from a biotechnological point of view. As subsequently shown, novel chemical and stereochemical properties found in examples of marine biocatalysts should be appended to the list of habitat related characteristics possessed by marine enzymes. Important examples have been found among oxidoreductases and carbohydrate active enzymes. As for the first class of enzymes, different stereoselectivity could be observed with respect to what is found in terrestrial counterparts adding flexibility to the often observed resistance to high salt concentration and/or organic solvent resistance. Biocatalysts for a potent chemical action on non-activated carbon atoms have been found in the marine context. Also, in these cases, substrate preferences and positions of functionalization could differ from known examples. Among enzymes acting on carbohydrates, the tendency for polyglycosylation is an interesting common quality of *Aplysia* α-glucosidase and other enzymes of marine origin and deserves in-depth examination. As far as lipid active hydrolases are concerned the case of the novel esterase from *Yarrowia lipolytica* CL180 for the enantioselective resolution of racemic ofloxacin propyl ester is very important. Other hydrolytic activities specific for different substrates include very interesting examples detailed above. The spectacular example of PatG, serving as a useful general biocatalyst for the cyclization of peptides, is remarkable and the great advantages of marine enzymes has also been demonstrated by studies on epoxide hydrolases possessing specific stereochemistry.

As demonstrated here, the enormous pool of biodiversity found in marine ecosystems is an excellent source for collecting an inventory of diverse biocatalysts.
